# AcceleRater: a web application for supervised learning of behavioral modes from acceleration measurements

**DOI:** 10.1186/s40462-014-0027-0

**Published:** 2014-12-25

**Authors:** Yehezkel S Resheff, Shay Rotics, Roi Harel, Orr Spiegel, Ran Nathan

**Affiliations:** Movement Ecology Laboratory, Department of Ecology, Evolution and Behavior, Alexander Silberman Institute of Life Sciences, The Hebrew University of Jerusalem, Jerusalem, Israel; Edmond and Lily Safra Center for Brain Sciences, The Hebrew University, Jerusalem, 91904 Israel; Present address: Department of Environmental Science & Policy, University of California at Davis, Davis, CA 95616 USA

**Keywords:** AcceleRater, Animal behavior, Biologging, Classification, Ethology, Movement ecology, Supervised learning, Tri-axial acceleration, Web application

## Abstract

**Background:**

The study of animal movement is experiencing rapid progress in recent years, forcefully driven by technological advancement. Biologgers with Acceleration (ACC) recordings are becoming increasingly popular in the fields of animal behavior and movement ecology, for estimating energy expenditure and identifying behavior, with prospects for other potential uses as well. Supervised learning of behavioral modes from acceleration data has shown promising results in many species, and for a diverse range of behaviors. However, broad implementation of this technique in movement ecology research has been limited due to technical difficulties and complicated analysis, deterring many practitioners from applying this approach. This highlights the need to develop a broadly applicable tool for classifying behavior from acceleration data.

**Description:**

Here we present a free-access python-based web application called AcceleRater, for rapidly training, visualizing and using models for supervised learning of behavioral modes from ACC measurements. We introduce AcceleRater, and illustrate its successful application for classifying vulture behavioral modes from acceleration data obtained from free-ranging vultures. The seven models offered in the AcceleRater application achieved overall accuracy of between 77.68% (Decision Tree) and 84.84% (Artificial Neural Network), with a mean overall accuracy of 81.51% and standard deviation of 3.95%. Notably, variation in performance was larger between behavioral modes than between models.

**Conclusions:**

AcceleRater provides the means to identify animal behavior, offering a user-friendly tool for ACC-based behavioral annotation, which will be dynamically upgraded and maintained.

**Electronic supplementary material:**

The online version of this article (doi:10.1186/s40462-014-0027-0) contains supplementary material, which is available to authorized users.

## Background

Movement ecology aims to unify organismal movement research and to aid in the development of a general theory of whole-organism movements [1]. The field has recently experienced a period of rapid growth in knowledge and insights [2], triggered by the advent of movement tracking tools and GPS devices in particular [3], as well as various methods of analyzing movement patterns [4]. These advances have motivated the development of integrative conceptual frameworks unifying cognitive, biomechanical, random and optimality paradigms to study movements of all kinds by all types of organisms [1]. Nevertheless, movement data, however accurate, are unlikely to suffice for inference on the links between behavioral, ecological, physiological, and evolutionary processes driving the movement of individuals, which have traditionally been studied in isolation in each of the movement research paradigms. Thus, promoting movement ecology research and the desirable unification across species and movement phenomena requires developing additional sensors and tools providing simultaneous information about the movement, energy expenditure and behavior of the focal organisms, and the environmental conditions they encounter *en route* [5].

To help bridge this gap, accelerometers were introduced as a means of identifying moment-to-moment behavioral modes [6] and estimating energy expenditure [7] of tagged animals. These sensors record body acceleration either in short bouts or continuously, along one, two or three orthogonal axes. Their output is used to infer behavior, most commonly through supervised machine learning techniques, and energy expenditure using the Overall Dynamic Body Acceleration (ODBA) or related metrics [7,8]. Combined with GPS recordings, acceleration sensors add fine scale information on the variation in animal’s behavior and energy expenditure in space and time (see [9] for a recent review). ACC-based analysis allows us to compute many measures of interest, including behavior-specific body posture, movement and activity budgets, measures of foraging effort, attempted food capture events, mortality detection, classifying behavioral modes and more [9]. These measures have facilitated movement-related research for a wide range of topics in ecology and animal behavior [5,9-11] as well as other fields of research such as animal conservation and welfare [10,12] and biomechanics [13,14].

An ACC dataset typically consists of anywhere between tens of thousands to millions of records, together with a small subset of hundreds or thousands of records corresponding to field observations which have known behavioral modes attached to them. A variety of machine learning algorithms have recently been applied for ACC-based supervised learning of behavioral modes [5,15-20]. These methods require a calibration set for ground-truthing, which associates behavioral classes to ACC measurements, by time-matching behavioral observations of tagged individuals to the recorded ACC. This calibration set is generally collected through field observations of free-ranging animals [5,9], but can also be obtained by observing animals in captivity [9,21]. In principle, the calibration dataset can also be generated from a biomechanical model, by generating the acceleration patterns expected in each behavioral mode using a model of an animal, though we are not aware of a published example of this alternative option. The entire calibration set, or its sub-set (called training set, see *cross validation* below), is used to learn how to classify ACC measurements to behavioral classes. An underlying assumption here is that during each measurement, the animal is engaged in a single behavioral mode. To assess classification performance, measures like accuracy, precision and recall are calculated, as illustrated in the *Results* section below. Typically, the calibration set constitutes only a (very) small sample of the recorded dataset; hence, in the final stage of ACC-based behavioral analysis, the classifier is used to assign behavioral modes to the whole dataset which may span the lifetime of many animals.

ACC-based behavioral data can inform “what” the study animal is doing in addition to the more conventional data on “where” the animal is located, acquired by the GPS units. However, in spite of this and the above-mentioned advantages of ACC data, many ecologists do not utilize this option even when they have acceleration sensors in their tracking devices. In part, this is due to the fact that some elusive species are very difficult to observe in order to obtain the above mentioned calibration set. However, in many other cases we believe that the computational procedures, and the technical challenges involved, deter researchers from using ACC-based behavioral data.

AcceleRater was developed to provide a user-friendly free-access tool for choosing, validating and using models for supervised learning of behavioral modes from ACC data. We hope that this tool will encourage the use of ACC-behavioral data with the promising insights it can provide.

## Implementation

AcceleRater is a python-based web application, using the sci-kit learn library [22] for fitting models and for most pre-processing operations. AcceleRater aims to facilitate broad use of ACC-based behavioral classification by including detailed explanations, a variety of models, model reconstruction options, alternative tests, and informative outputs, and by allowing the user to control many aspects of the processing, while setting typical values as default options.

### Input data format

AcceleRater requires the user to prepare the input data file in advance. Although the package can be designed to obtain data directly from default output formats of some commercially-available ACC loggers, supervised methods require coupling ACC records with observed behaviors, necessitating some processing of the default ACC file in any case. In addition, accelerometers provide hardware-unit-specific measurements which require calibration for each tag, thereby typically requiring another pre-processing stage. Furthermore, the raw ACC data can be measured along one, two or three axes, and some devices provide some summary statistics rather than the raw data (see Additional file 1: Table S3 in supplementary material). To accommodate these needs and varieties, the user first indicates some basic attributes of the input dataset, including contents (summary statistics or raw data), and, for raw data files, the number of axes (1, 2 or 3) for which ACC data was measured. For any selection, the user is offered several input file structures, all should be formatted as comma separated values (csv) files, with ACC measurements in rows, and behavior labels in the last column. Example data files can be found on the *demo* page of the application website.

### The computing and feature selection protocol

1. Selecting and calculating summary statistics: For input files with raw ACC data, the user needs to select summary statistics to be calculated from the raw data. The list of summary statistics currently implemented in the program is given in Additional file 2: Table S1 (supplementary material). Additional statistics will be added upon user requests.
2. Processing summary statistics: The program calculates and then normalizes (to zero mean and unit standard deviation) all summary statistics selected in step (1).
3. Selecting the cross validation method: Cross-validation methods [23] separate the calibration dataset to training and validation subsets, the former is used to build the model, and the latter enables the user to quantify how well the calibrated model matches independent observations. We offer three options for performing validation: (a) *k*-fold cross-validation, the dataset is randomly split into *k* equal-size parts, *k*-1 parts are used for training and 1 for validation. The procedure is repeated *k* times until all parts have been used for validation; (b) a special case of (a), with *k* = 2, known as train-split method. This is the fastest and most commonly used option, taken here as the default; (c) another special case of (a), known as Leave-One-Out method, with *k* = n where *n* is the number of labeled samples available. For large *n*, this option is computationally expensive, as well as unnecessary; hence the use of this option should be limited to rather small datasets (currently hundreds of samples).
4. Selecting and computing the models, and presentation of the results: the user selects one or more classifiers, listed in Table 1 and briefly outlined in (Additional file 3: Table S2. Once the selection is completed, the normalized statistics are fed into the chosen classifiers. Then, the cross-validation and some other results are displayed in the form of summary tables, confusion matrices, and accuracy, recall and precision tables (see examples in *Results* section below).
5. Using the calibrated model to label new data, see *“Labeling new data”* below.

**Table 1:** **A list of classification models currently implemented in AcceleRater, with representative published applications for classifying animal behavior**

**Model**	**Sources**
Artificial Neural Network (ANN)	[5]
Decision tree	[5]
Linear support vector machine (L-SVM)	[5]
Linear/Quadratic Discriminant Analysis (LDA/QDA)	[5,16,17]
Nearest neighbors	[19]
Radial basis function kernel for support vector machine (RBF-SVM)	This paper
Random forest	[5,16,20]

### Using the application

The minimal requirement is to upload the labeled (ground-truthed) ACC data file and run the program with default selection of its various options. Alternatively, the user can select the summary statistics, the cross validation method and the models.

### Main features

*Manual* - the manual contains an extensive documentation of the application, and should be referred to for further information.

*Upload form* - The “gateway” to the application. See *Input data format* above.

*Models view* - Here the models are summarized. This view contains:

- A page for each model with a confusion matrix in both graphical and tabular form, as well as overall accuracy and recall/precision/accuracy tables.
- A graph comparing the overall accuracy for each of the models
- A precision-recall graph comparing the models.
- A table containing the specific accuracy/recall/precision for every behavior in each model. This may be important when some of the behaviors are of more significance for the purpose at hand, and it is therefore desirable to select a model that does best on these behaviors.

*Labeling new data* – Beyond its use for assessing the feasibility and reliability of ACC-based behavioral classification for a given dataset, arguably the main purpose of using AcceleRater is to annotate (label) a large set of ACC recordings for which behavioral information is not available. The user should upload a file for annotation in an acceptable format (see *Input data format* above). The output csv file is the same as the input file, with an added last column providing the assigned behavioral labels.

*Annotating a trajectory on a map* – To visualize a trajectory of an animal on a map, annotated with the ACC-based behavioral labels, the program allows the user to upload a raw data file with both location (e.g. from GPS) and ACC data. The trajectory is then shown on a Google Map with different colors indicating different behaviors. Currently, the program supports raw data file format of only one manufacturer (E-Obs GmbH; Munich, Germany), but other formats will be implemented upon users’ requests.

## Results

**Figure 1:**
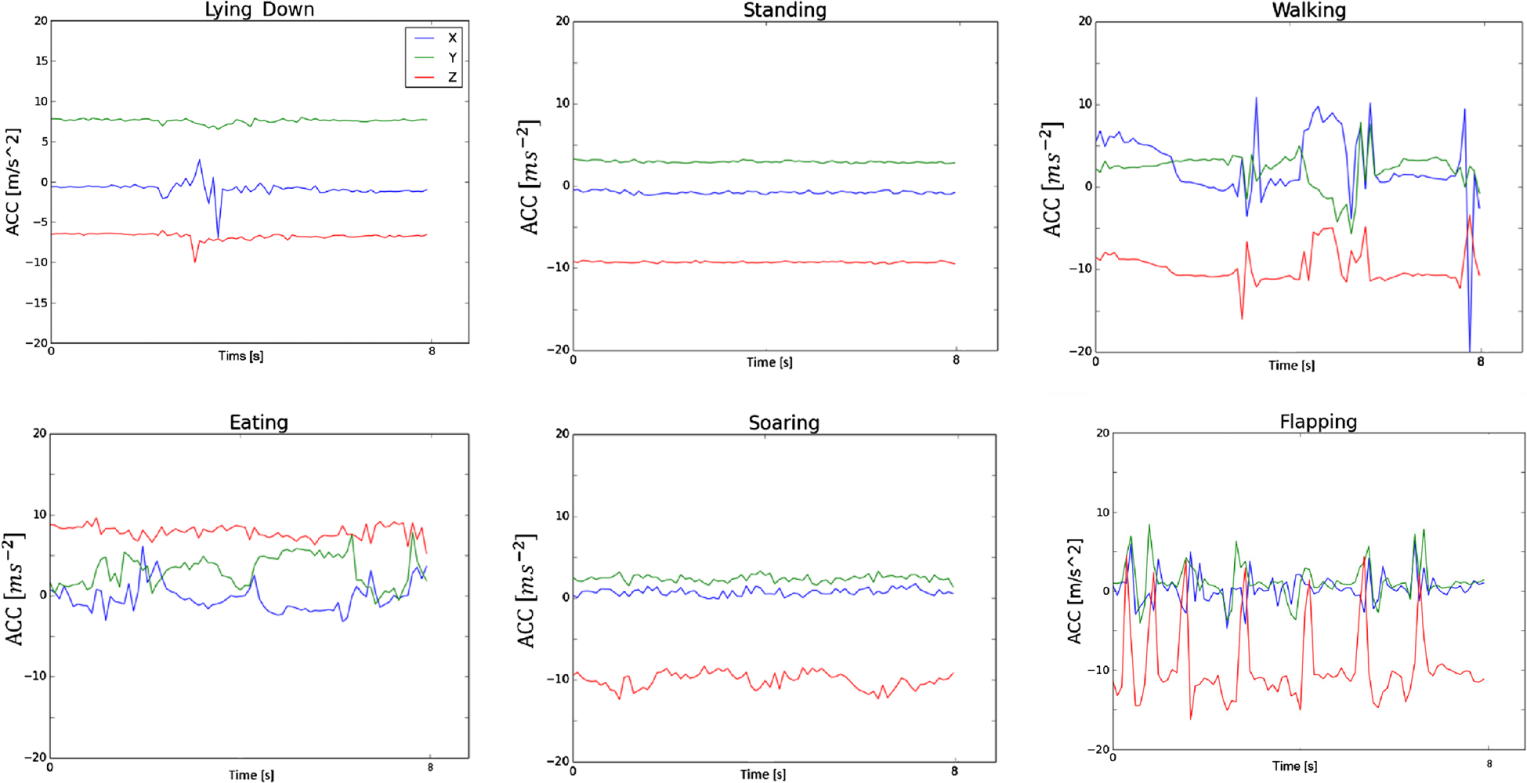
**Representative acceleration plots for the six different behavioral modes obtained by AcceleRater from the vulture dataset.** Each plot represents a single behavioral segment. Acceleration was sampled at 10Hz per axis.

To test AcceleRater, we used ACC data collected by E-Obs transmitters on Griffon Vultures (*Gyps fulvus*). Acceleration was measured at 10Hz per axis and segments corresponding to single behavioral modes were obtained by field observations. For more details on this dataset see Refs. [5] and [11]. We used a dataset comprising of 488 samples and 6 behavior classes: Lying down (3.5%), Standing (43.6%), Walking (13.7%), Eating (22.3%), Soaring (6.6%), Flapping (10.2%). Typical acceleration signatures of the different behaviors are shown in Figure 1.

**Figure 2:**
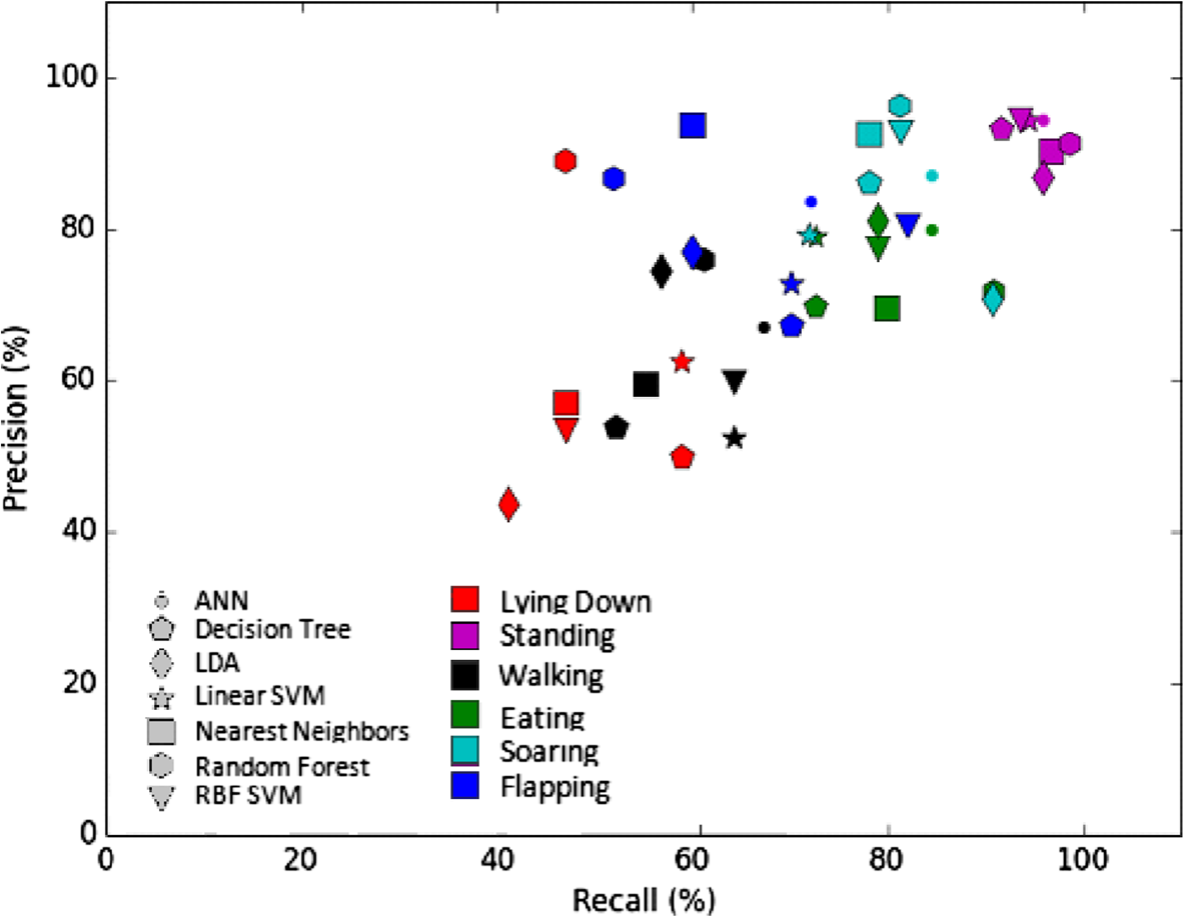
**Precision-recall plot generated by accelerater for the vulture dataset (see Additional file** **4** **: Table S4).**

The main variation in the overall accuracy (Table 2), and in specific accuracy, precision and recall of assignment in the cross validation tests was attributed to different behaviors rather than different models (Additional file 4: Table S4, Figure 2). The specific accuracy of assignment to a particular behavior – the probability of a sample in the test-set to be assigned correctly to the specific behavior (True Positive; TP) or to another behavior (True Negative; TN) – was on average 91-94% for each model and 90-97% for each behavior across models (Additional file 4: Table S4b). The precision of assignment – the probability that an assigned behavior in the test-set is indeed this particular behavior – was medium to high (78-85%) for the different models, very high (92%) for Standing, high (80-86%) for both flying types and lower (59-75%) for the other three behaviors (Additional file 4: Table S4c). The recall – the probability that a sample with a particular behavior in the test-set will be correctly classified as this behavior – was relatively high (77-85%) for the different models, extremely high (95%) on average for Standing (the most common behavior in the training set), medium (80%) for Soaring and for Eating and lower (51-66%) for Walking, Flapping and Lying down (Additional file 4: Table S4d). These results are effectively summarized by the Precision-Recall plot (Figure 2). Note that overall accuracy, recall and precision of the ANN model were slightly better compared to other models (Table 2 & Additional file 4: Table S4), but in general all models preformed reasonably well (Table 2).

**Table 2:** **Model accuracy**

**Model name**	**% correct**	**Std**
ANN	84.84	2.76
Decision tree	77.68	5.76
LDA	80.75	4.89
Linear SVM	80.13	4.18
Nearest neighbors	80.54	3.18
Random forest	84.02	2.98
RBF SVM	82.58	3.91
**Mean**	**81.51**	**3.95**

Standard deviation computed using a 10-fold cross validation procedure.

## Discussion

The use of accelerometers in movement ecology has become popular in recent years, partly due to improvements in the underlying technologies and the advent of analysis tools [5]. Nevertheless, the non-trivial process of supervised learning of behavioral modes from acceleration data has hindered much more widespread use of this technique. Towards this end, we developed AcceleRater as a specialized web application for rapidly training, visualizing and using models for supervised learning of behavior modes from ACC measurements.

AccleRater was tested with 488 ACC segments collected by GPS-ACC transmitters (E-Obs GmbH; Munich, Germany) on Griffon Vultures (*Gyps fulvus*). We ran stratified random selection on a roughly twofold larger dataset [5] to reduce over-dominance of commonly observed behaviors. For this dataset, we found that model selection is a less critical consideration, compared to highly variable results for different behaviors. This might complicate analyses requiring reliable classification of many behaviors, whereas studies focusing a single or few behaviors could choose the best fitted model for their study system. AcceleRater yielded comparable results to those we previously reported for this dataset [5], extending our previous analysis by including additional models (RBF-SVM) and more informative output (e.g., precision and recall, rather than only accuracy). Most importantly, whereas previous contributions from our group as well as others [5,11,9,15,20] have provided guidelines for such analyses, AcceleRater practically implements and extends these guidelines, making this technique available for a broad range of users. It allows a thorough analysis that can be carried out quickly and effectively, yielding informative results within minutes.

### Usage considerations

The online nature of the application requires transfer of data files over the internet. This inherently limits the size of the data files to be labeled. When labeling a large dataset with this application, the data should be broken down into manageable size parts, with ≤100,000 rows each.

### Future work

The supervised learning framework is based upon observations being sampled from the distribution of the process in question. This sample, however, might not adequately reflect the true distribution of these behaviors throughout the time frame relevant to the research question, due to practical constraints of field observations, for example. Consequently, behavioral modes that are rare in the observation sample, and as such discarded or have weak classifiers, may in fact be more common and/or more influential for the study system. This concern motivates refinement of field observations on the one hand, and development of data-driven methods for unsupervised learning of behavior modes from ACC data on the other hand.

The segmentation of movement tracks has been identified as one of the greatest methodological challenges in movement ecology research [1]. By providing behavioral information highly relevant for distinguishing different movement phases, ACC-based behavioral classification can facilitate addressing this challenge [20]. AcceleRater can therefore be extended to suggest segmentation pattern for movement tracks based on behavioral classification.

A key limitation of AcceleRater, like other web applications, is the need to upload and download large data files for labeling after a model is trained and chosen. This limitation might prohibit the use of the application on large datasets, with many millions of data points. We plan to address this limitation in future versions by allowing the user to select a model using the web application, and then download a stand-alone program configured to classify new data using the selected model offline, on the user’s computer.

## Conclusions

We present here a new tool, AcceleRater, allowing fast and intuitive tool for ACC-based behavioral classification, designed to be both flexible and general, with user-friendly interface and informative results displayed in tables and graphs. We demonstrate high performance of this tool in classifying behaviors of free-ranging birds. We encourage broad use and foresee further developments of AcceleRater for advancing more informative analysis of the ecology and behavior of animals in the wild.

## Availability and requirements

**Project name**: AcceleRater.

**Project home page**: http://accapp.move-ecol-minerva.huji.ac.il/.

**Operating system(s)**: Platform independent.

**Programming language**: Python, JavaScript.

**License**: The program was developed by YR and owned by the Minerva Center for Movement Ecology. We encourage its free use, no permission or license is required. The current paper should be cited in resulting publications.

**Any restrictions to use by non-academics**: none.

## Additional files

Additional file 1: Table S3. Examples of ACC tag manufacturers and the type of supported output. Additional file 2: Table S1. The statistics computed by the application. Additional file 3: Table S2. The classification models currently (April 2014) implemented in AcceleRater. For updates, please visit the web site of the Minerva Center for Movement Ecology (http://accapp.move-ecol-minerva.huji.ac.il/). Additional file 4: Table S4. Recall, Precision and Accuracy for each of the models and behaviors.

## Abbreviations

ACC: Acceleration
ANN: Artificial neural network
ODBA: Overall dynamic body acceleration
RBF-SVM: Radial basis function SVM
SVM: Support vector machine
