# Supplementary material for: AcceleRater: a web application for supervised learning of behavioral modes from acceleration measurements
Source: Mov Ecol. 2014 Dec 25;2(1):27. doi: 10.1186/s40462-014-0027-0 (PMC4337760; doi:10.1186/s40462-014-0027-0)
Supplement: Additional file 1: Table S3. — Examples of ACC tag manufacturers and the type of supported output. [file 40462_2014_27_MOESM1_ESM.doc]

Table S3. Examples of ACC tag manufacturers and the type of supported output.

| Tag name | Manufacturer | Output type | link |
| --- | --- | --- | --- |
| UvA-BiTS | University of Amsterdam | Raw ACC data | <http://www.uva-bits.nl/system/> |
| e-obs | e-obs | Raw ACC data | <http://www.e-obs.de/service.html> |
| AXY-2 | Technosmart | Raw ACC data | <http://www.technosmart.eu/> |
| Vectronic | Vectronic | Summary statistics | <http://www.vectronic-aerospace.com/> |
