# Supplementary material for: AcceleRater: a web application for supervised learning of behavioral modes from acceleration measurements
Source: Mov Ecol. 2014 Dec 25;2(1):27. doi: 10.1186/s40462-014-0027-0 (PMC4337760; doi:10.1186/s40462-014-0027-0)
Supplement: Additional file 2: Table S1. — The statistics computed by the application. [file 40462_2014_27_MOESM2_ESM.doc]

Table S1. The statistics computed by the application.

| Statistic | Notes/Explanation |
| --- | --- |
| Mean | Preformed on each axis separately. |
| Standard deviation (Std) |
| Skewness |
| Kurtosis |
| Max |
| Min |
| Norm | The vector norm of the ACC sample. This is normalized by the length of the sample, to allow a meaningful use of varying length samples. |
| Cov | Covariance between pairs of axes. |
| r | Pearson's correlation between every two axes. |
| mean-diff | The mean difference between every two axes |
| Std-diff | The standard deviation of the difference between every two axes. |
| DBA | Dynamic body acceleration by axis? |
| ODBA | Overall dynamic body acceleration. The sum over the axes of the DBA. |
| Wave-amplitude | The average difference between consecutive local minima and maxima. |
| Line crossings | The number of times each two axes cross over each-other. |
| 25% | The 25, 50 and 75 percentile of each axis, respectively. |
| 50% |
| 75% |
