# Supplementary material for: AcceleRater: a web application for supervised learning of behavioral modes from acceleration measurements
Source: Mov Ecol. 2014 Dec 25;2(1):27. doi: 10.1186/s40462-014-0027-0 (PMC4337760; doi:10.1186/s40462-014-0027-0)
Supplement: Additional file 3: Table S2. — The classification models currently (April 2014) implemented in AcceleRater. For updates, please visit the web site of the Minerva Center for Movement Ecology (http://accapp.move-ecol-minerva.huji.ac.il/). [file 40462_2014_27_MOESM3_ESM.doc]

Table S2. The classification models currently (April 2014) implemented in AcceleRater. For updates, please visit the web site of the Minerva Center for Movement Ecology (http://accapp.move-ecol-minerva.huji.ac.il/)

| Model | Overview |
| --- | --- |
| Artificial Neural Network (ANN) | A biologically inspired method for learning complex relations between inputs and outputs, capable of acting as a universal function approximator. |
| Decision Tree | A branching multi-stage decision rule, using only one variable at each stage. |
| Linear support vector machine (L-SVM) | The basic version of the Support Vector Machine (SVM) classifier produces a binary classification by computing the maximum margin separating hyperplane between the two classes (The hyperplane that separates the two classes, and has the maximal distance from the data point closest to it). This extension to more than two classes computes the binary classifier on each pair and then use a majority vote. |
| Linear/Quadratic Discriminant Analysis (LDA/QDA) | LDA reduces the dimensionality of the data by computing the one-dimensional projection which maximizing the variance between the classes while minimizing the variance within the classes. LDA is a parametric method that assumes homoscadastic Gaussian distributions of classes. QDA relaxes this setting to the heteroscadastic case. |
| Nearest Neighbors | Each new data point is classified according to a vote between the k labeled points nearest to it. This model relies on the proximity assumption that nearby points tend to belong to the same class. |
| Radical basis function kernel for support vector machine (RBL-SVM) | An extension of the SVM algorithm, where the data is implicitly projected onto an infinite dimension space before the hyperplane is computed. |
| Random Forest | An ensemble of decision trees. The output is decided using a majority vote. |
