# Supplementary material for: AcceleRater: a web application for supervised learning of behavioral modes from acceleration measurements
Source: Mov Ecol. 2014 Dec 25;2(1):27. doi: 10.1186/s40462-014-0027-0 (PMC4337760; doi:10.1186/s40462-014-0027-0)
Supplement: Additional file 4: Table S4. — Recall, Precision and Accuracy for each of the models and behaviors. [file 40462_2014_27_MOESM4_ESM.docx]

| **a.** Definitions (illustrated for the "Eating" behavior)  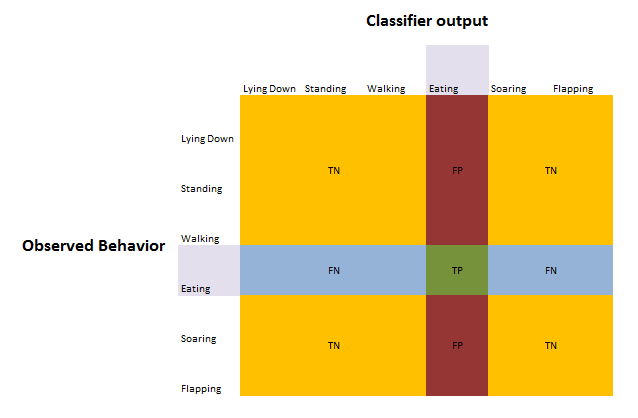 | **c.** Accuracy: $\frac{TP+TN}{TP+TN+FP+FN}$   \|  \| Lying Down \| Standing \| Walking \| Eating \| Soaring \| Flapping \| **weighted average** \| \| --- \| --- \| --- \| --- \| --- \| --- \| --- \| --- \| \| ANN \| 97 \| 96 \| 91 \| 92 \| 98 \| 96 \| **94** \| \| Decision Tree \| 97 \| 93 \| 87 \| 87 \| 98 \| 93 \| **92** \| \| LDA \| 96 \| 92 \| 91 \| 91 \| 97 \| 94 \| **92** \| \| Linear SVM \| 97 \| 95 \| 87 \| 90 \| 97 \| 94 \| **93** \| \| Nearest Neighbors \| 97 \| 94 \| 89 \| 88 \| 98 \| 95 \| **92** \| \| Random Forest \| 98 \| 95 \| 92 \| 90 \| 99 \| 94 \| **94** \| \| RBF SVM \| 97 \| 95 \| 89 \| 90 \| 98 \| 96 \| **93** \| \| **mean** \| **97** \| **94** \| **90** \| **90** \| **98** \| **95** \| **93** \| |
| --- | --- | --- | --- | --- | --- | --- | --- | --- | --- | --- | --- | --- | --- | --- | --- | --- | --- | --- | --- | --- | --- | --- | --- | --- | --- | --- | --- | --- | --- | --- | --- | --- | --- | --- | --- | --- | --- | --- | --- | --- | --- | --- | --- | --- | --- | --- | --- | --- | --- | --- | --- | --- | --- | --- | --- | --- | --- | --- | --- | --- | --- | --- | --- | --- | --- | --- | --- | --- | --- | --- | --- | --- | --- |
| **b.** Precision: $\frac{TP}{TP+FP}$   \|  \| Lying Down \| Standing \| Walking \| Eating \| Soaring \| Flapping \| **weighted average** \| \| --- \| --- \| --- \| --- \| --- \| --- \| --- \| --- \| \| ANN \| 63 \| 94 \| 67 \| 80 \| 87 \| 84 \| **85** \| \| Decision Tree \| 50 \| 93 \| 54 \| 70 \| 86 \| 67 \| **78** \| \| LDA \| 44 \| 87 \| 75 \| 81 \| 71 \| 77 \| **80** \| \| Linear SVM \| 63 \| 94 \| 52 \| 79 \| 79 \| 73 \| **81** \| \| Nearest Neighbors \| 57 \| 90 \| 60 \| 70 \| 93 \| 94 \| **81** \| \| Random Forest \| 89 \| 91 \| 76 \| 72 \| 96 \| 87 \| **85** \| \| RBF SVM \| 53 \| 94 \| 60 \| 77 \| 93 \| 80 \| **83** \| \| **mean** \| **60** \| **92** \| **63** \| **76** \| **86** \| **80** \| **82** \| | **d.** Recall: $\frac{TP}{TP+FN}$   \|  \| Lying Down \| Standing \| Walking \| Eating \| Soaring \| Flapping \| **weighted average** \| \| --- \| --- \| --- \| --- \| --- \| --- \| --- \| --- \| \| ANN \| 59 \| 96 \| 67 \| 84 \| 84 \| 72 \| **85** \| \| Decision Tree \| 59 \| 92 \| 52 \| 72 \| 78 \| 70 \| **78** \| \| LDA \| 41 \| 96 \| 57 \| 79 \| 91 \| 60 \| **81** \| \| Linear SVM \| 59 \| 94 \| 64 \| 72 \| 72 \| 70 \| **80** \| \| Nearest Neighbors \| 47 \| 97 \| 55 \| 80 \| 78 \| 60 \| **81** \| \| Random Forest \| 47 \| 99 \| 61 \| 91 \| 81 \| 52 \| **84** \| \| RBF SVM \| 47 \| 93 \| 64 \| 79 \| 81 \| 82 \| **83** \| \| **mean** \| **51** \| **95** \| **60** \| **80** \| **81** \| **67** \| **82** \| |

Table 3. Recall, Precision and Accuracy for each of the models and behaviors.
